# How to avoid bulky knots when using loop sutures

**DOI:** 10.1308/003588413X13511609957056g

**Published:** 2013-01

**Authors:** R Durai, T Oke

**Affiliations:** Woolwich, London, SE18 4QH

## Background

Monofilament absorbable and non-absorbable sutures are commonly used for closure of the anterior abdominal wall during laparotomy. Monofilament sutures have a memory and, as a result, have a poor knot holding capacity: 5–6 throws are required to make the knot secure. The resultant bulky knot can be difficult to bury and, when it is not buried properly, the patient may suffer from pain, a palpable lump or a stitch sinus resulting in a persistent discharge. We report a simple method that can be used to reduce the bulkiness of the knot.

## Technique

**Figure 1:**
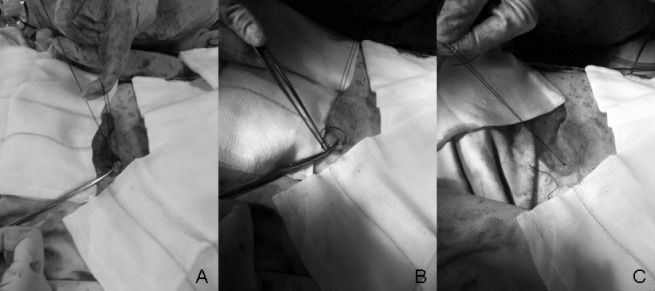
Figure 1 A) Before feeding the suture through the loop B) Passing the suture through the loop C) Completed knot with 6 throws

This technique is used for the closure of pfannenstiel and transverse incisions where one suture is sufficient enough to close the entire abdominal wall. Once the abdominal wall wound is closed by one continuous loop suture, instead of making an Aberdeen knot, one of the two threads attached to the needle is cut and passed under the loop of the thread. The two individual ends of the thread are tied together, the excess length of the free thread is cut off and the suture attached to the needle is passed through the middle of the suture line, in the process the knot is buried (Fig 1). When two loop sutures are used to close a midline laparotomy wound (one from each end), one of the needles is cut off when the wound is fully approximated. The free thread is tied to the thread attached to the opposite needle. The excess length of the free thread is cut off and the knot is buried in the suture line; the 4 threads of suture including the remaining needle are cut flush on the wound.

## Discussion

The main purpose of this technique is to make the knot less bulky and easy to bury. With several years of use, we have not encountered any stitch sinuses from this method. It is simple, quick, easy and reproducible without any difficulty. This technique reduces the bulk of the knot and enables the knot to be completely buried in the wound.

